# Cost-effectiveness of counselling, graded-exercise and usual care for chronic fatigue: evidence from a randomised trial in primary care

**DOI:** 10.1186/1472-6963-12-264

**Published:** 2012-08-20

**Authors:** Ramon Sabes-Figuera, Paul McCrone, Mike Hurley, Michael King, Ana Nora Donaldson, Leone Ridsdale

**Affiliations:** 1 Centre for the Economics of Mental and Physical Health (CEMPH), Institute of Psychiatry, King’s College London, London, UK; 2 Academic Department of Physiotherapy, Health and Social Care Research Division, King’s College London, London, UK; 3 Department of Mental Health Sciences, Hampstead Campus, University College London, London, UK; 4 Dental Institute, King’s College London, London, UK; 5 Unit of Neurology and General Practice, Department of Clinical Neuroscience, Institute of Psychiatry, King’s College London, London, UK

**Keywords:** Chronic fatigue, Cost-effectiveness, Randomised controlled trial

## Abstract

**Background:**

Fatigue is common and has been shown to result in high economic costs to society. The aim of this study is to compare the cost-effectiveness of two active therapies, graded-exercise (GET) and counselling (COUN) with usual care plus a self-help booklet (BUC) for people presenting with chronic fatigue.

**Methods:**

A randomised controlled trial was conducted with participants consulting for fatigue of over three months’ duration recruited from 31 general practices in South East England and allocated to one of three arms. Outcomes and use of services were assessed at 6-month follow-up. The main outcome measure used in the economic evaluation was clinically significant improvements in fatigue, measured using the Chalder fatigue scale. Cost-effectiveness was assessed using the net-benefit approach and cost-effectiveness acceptability curves.

**Results:**

Full economic and outcome data at six months were available for 163 participants; GET = 51, COUN = 58 and BUC = 54. Those receiving the active therapies (GET and COUN) had more contacts with care professionals and therefore higher costs, these differences being statistically significant. COUN was more expensive and less effective than the other two therapies. The incremental cost-effectiveness ratio of GET compared to BUC was equal to £987 per unit of clinically significant improvement. However, there was much uncertainty around this result.

**Conclusion:**

This study does not provide a clear recommendation about which therapeutic option to adopt, based on efficiency, for patients with chronic fatigue. It suggests that COUN is *not* cost-effective, but it is unclear whether GET represents value for money compared to BUC.

Clinical Trial Registration number at ISRCTN register: 72136156

## Background

Fatigue has been shown to result in high economic costs to society, especially because of its impact on employment and the need for families and friends to spend time caring for the individual [1,2]. Therapies including cognitive behaviour therapy (CBT), graded exercise therapy (GET) and counselling, have been associated with reduced fatigue in primary care patients six months later [3,4]. However, economic evaluations of these interventions have reported inconclusive results, with considerable uncertainty regarding cost-effectiveness estimates [5-7].

The aim of this paper is to compare the cost-effectiveness of two of these active therapies, GET and counselling (COUN) with usual GP care plus a self-help booklet (BUC). The perspective taken is that of the health service.

## Method

### Sample, setting and interventions

Exclusion and inclusion criteria of the clinical trial have been described elsewhere [2,8]. Briefly, participants were patients presenting to their general practitioners (GPs) complaining of fatigue with a duration of more than three months. They were randomised to one of the three therapies considered: GET, COUN and usual GP care plus a self-help booklet (BUC). For GET and COUN, participants were offered eight sessions of treatment at two-week intervals at their local general practice, followed by two telephone calls one month apart. For GET, the durations of the initial and subsequent sessions were 45 and 30 minutes respectively and it consisted of supervised exercise by physiotherapists, adapted to each patient’s current physical capacity, which is gradually increased in duration according to a protocol designed for patients with chronic fatigue. COUN patients were offered 60 minute face-to-face sessions with a counsellor registered with the British Association for Counselling and Psychotherapy (BACP). The counselling style used in this study followed the Rogerian client centred, non-directive format that encouraged the patient to talk through difficulties, and reflect on their experiences and thoughts in order to understand themselves better, to arrive at alternative understandings, to uncover the links between current distress and past experience, and to provide the conditions for growth and healing.

### Outcomes

Assessments were made at baseline with follow-up at six and twelve months. The primary clinical outcome was the Chalder fatigue scale [9], which consists of 13 items assessed using Likert scales (0,1,2,3) producing a total score ranging between 0 and 33. For the purpose of the economic evaluation we calculated the amount of clinically significant change by the six-month follow-up given that use of services data was not available for the twelve months follow-up. This was obtained by dividing the actual change in the Chalder fatigue scale total score by four. Thus a value of one in the change in fatigue outcome corresponds to a difference of four in the original Chalder fatigue scale, assuming that a change of that magnitude was clinically significant (CSI) [6]. This was based in a consensus reached by clinicians in a previous trial [10].

### Service use and costs

The Client Service Receipt Inventory [11] was used to retrospectively record service use for six months at the follow-up time point and at baseline. Patients were asked to provide details of health and social care services used (including number of contacts and, where appropriate, the average duration). Services included primary and secondary healthcare contacts, complementary healthcare, social care and medication (only anti-depressant, anti-anxiety and sleeping medication). These service use data were combined with appropriate unit costs for 2006/2007 obtained from national sources [12-14]. The unit costs of complementary and alternative therapies were obtained from other publications and sources [15-17]. The costs of different medicines were obtained from the British National Formulary for the year 2006 [18]. For GET and counselling the number of sessions attended by each participant was recorded, assuming an average duration of the sessions as previously stated. A nominal figure of £5 per BUC patient was used to represent the cost of the booklet used to enhance usual care.

### Analysis

Analyses were carried out for those patients for whom we had complete data in terms of clinical outcomes and cost at the six-month follow–up. Multiple regression was used to adjust for the following baseline characteristics in all tests of differences in costs and clinical outcomes: gender, age, whether the patient lived alone, whether the patient had dependants, symptom level, level of depression, level of anxiety, level of social functioning, baseline fatigue score, number of months since chronic fatigue began, and baseline health care cost. Regression analysis using cost data frequently results in non-normally distributed residuals. Therefore, bootstrapping was used which involves resampling with replacement from the original sample a sufficiently large number of times in order to approximate the distribution of the population from which the original data were drawn [19]. In our analyses, 1000 samples were generated. Cost-effectiveness was then explored through the calculation of incremental cost-effectiveness ratios (ICER), defined as the difference in mean costs divided by difference in mean effects [20]. Bootstrapped joint distributions of incremental mean costs and effects for the treatments was then used to calculate the probability that each of the treatments is the optimal choice, subject to a range of possible maximum values (ceiling ratio) that a decision-maker might be willing to pay for a unit improvement in outcome. Cost-effectiveness acceptability curves are presented by plotting these probabilities for a range of possible values of the ceiling ratio [21]. These curves incorporate the uncertainty that exists around the estimates of expected costs and expected effects associated with the interventions [22].

### Ethical approval

Multi Centre Research Ethics Committee (MREC) approval; West MidlandsMREC/02/7/71. Local approval; Lambeth, Southwark and Lewisham Primary Care Trusts RDLSLG 142.

## Results

**Figure 1:**
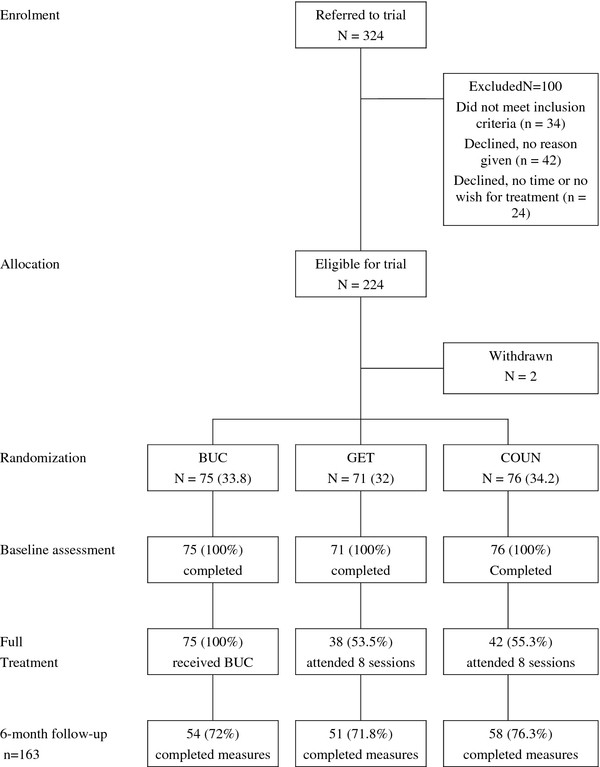
The flow of participants.

Of the 222 individuals who were randomised, full economic and outcome data at six-months follow-up were available for 163 participants (73%) (Figure 1. The Flow of Participants). Most of them were women (81%) with an average age of 41 years and a median duration of fatigue of two years. There were no significant statistical differences in clinical variables at baseline between individuals with complete data at six months and those without. However, the former were older and had a lower average duration of fatigue. The probability of not having completed data at six months did not depend on treatment arm assigned.

Table 1 details the use of resources for each treatment group. Participants receiving the active therapies had more contacts with care professionals than the usual care participants, both in the percentage of those who used these services and in the number of contacts. COUN patients had a slightly higher use of services than the GET patients. The cost of services reflects these service use differences (Table 1). The difference in the intervention costs was due to a more expensive cost of counselling sessions as a result of their longer duration. Table 1 also shows average Chalder scores at baseline and at six months. The improvement over that period is almost identical for the BUC and COUN groups, while those patients receiving GET, on average, had a slightly greater improvement.

**Table 1:** Average use of services, average costs (£ 2006/07) and average Chalder fatigue scale score

	**BUC (N = 54)**			**GET (N = 51)**			**COUN (N = 58)**		
**Professional**	**%**^**1**^	**Contacts**^**2**^**(sd)**	**Cost (£)**	**%**^**1**^	**Contacts**^**2**^**(sd)**	**Cost (£)**	**%**^**1**^	**Contacts**^**2**^**(sd)**	**Cost (£)**
GP	70	3.1 (1.91)	102.9	84	3.5 (3.20)	140.6	83	3.9 (2.46)	154.7
Other doctor	28	1.6 (0.74)	29.8	29	3.1 (2.34)	59.0	48	1.8 (1.16)	67.4
Neurologist	2	1.0 (−)	2.9	6	1.3 (0.58)	12.2	5	3.0 (2.00)	24.2
Psychiatrist	0	.	0.0	8	2.8 (1.71)	30.2	10	3.2 (2.48)	45.9
Nurse	24	1.2 (0.38)	2.2	24	1.3 (0.62)	2.4	31	2.3 (4.23)	5.7
Other care professional	22	6.6 (5.87)	49.2	35	4.6 (4.79)	44.9	22	4.5 (5.08)	33.7
Homeopath/ herbalist	4	4.5 (2.12)	9.1	8	3.5 (3.11)	15.0	9	6.4 (5.22)	30.2
Medication	33	--	11.9	31	--	13.3	29	--	12.0
Therapy	--		5.0	--	6.7 (2.4)	156.1	--	6.8 (2.3)	277.1
Total cost	212.9			473.6			650.8		
Chalder score Baseline	23.85			24.67			24.78		
Chalder score 6 months	15.30			14.61			16.16		
Improvement	8.56			10.06			8.62		

*GET* = graded exercise therapy, *BUC* = booklet plus usual care, *COUN* = counselling,

1. percentage of participants in each treatment group using the service. 2. average number ofcontacts for those who at least had one.

**Figure 2:**
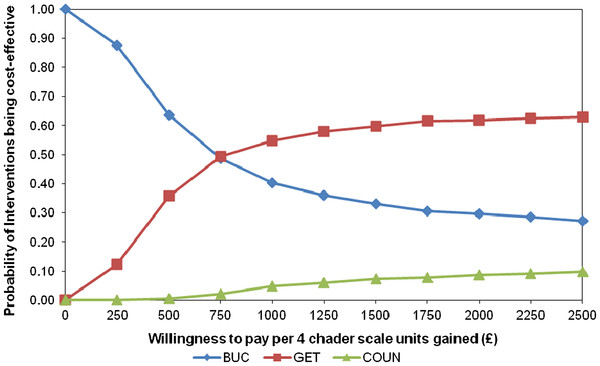
Cost-effectiveness acceptability curve of interventions.

Comparisons of total costs and outcomes between the three groups are shown in Table 2. The adjusted differences in costs and outcomes obtained between the three treatment arms show that COUN is significantly more expensive and non-significantly less effective (based on the Chalder fatigue scale) than the other two interventions. In economic terms COUN is ‘dominated’ by GET and BUC. GET is more expensive than BUC and more effective (albeit not significantly). The incremental cost-effectiveness ratio for the comparison of these two interventions is £987 per unit of CSI (gain of four points in the Chalder score) obtained as a result of GET. The uncertainty associated with this result is indicated in the cost-effectiveness acceptability curve (Figure 2. Cost-effectiveness acceptability curve of interventions evaluated.), where the probabilities of the interventions being cost effective are shown for different monetary values assigned to a gain of a unit of outcome (CSI). If this monetary value or willingness to pay for a CSI is equal to £1000, there is a 55% chance that GET is the most cost- effective option and just a 5% that COUN is the most cost-effective option. When the value placed on a CSI is equal to £2500, the probability that GET is the most cost-effective option reaches 63%. Analyses beyond this value (up to a willingness to pay of £100,000 for a CSI) found that the likelihood of GET being the intervention more cost-effective did not rise above 65% (and the value for COUN did not reach 17%).

**Table 2:** **Adjusted**^**1**^**differences in costs and outcomes and incremental cost-effectiveness ratios**

	**GET vs BUC**		**COUN vs BUC**		**GET vs COUN**	
	**Difference (95%CI)**	**p**	**Difference (95%CI)**	**p**	**Difference (95%CI)**	**p**
Costs differences	261(141 to 382)	<0.001	423 (288 to 559)	<0.001	−202 (−362 to −43)	0.013
Chalder improvements differences	1.1 (−2.3 to 4.4)	0.530	−0.1 (−3.1 to 2.9)	0.969	0.7 (2.6 to −4.0)	0.663
ICER per CSI	987		COUN dominated strategy		COUN dominated strategy	

*GET* = graded exercise therapy, *BUC* = booklet plus usual care, *COUN* = counselling, *ICER* = incremental cost-effectiveness ratio, *CSI* = clinically significant improvement.

1. adjusted by the following baseline characteristics: gender, age, whether the patient lived alone, whether the patient had dependants, symptom level, level of depression, level of anxiety, level of social functioning, baseline fatigue score, number of months since chronic fatigue began, and baseline health care cost.

## Discussion

Previous studies have shown that the majority of costs associated with chronic fatigue are hidden or indirect, such as informal care and lost productivity [2]. Nevertheless, in the current context of cost containment and efficiency savings, a narrower focus on healthcare costs as the one adopted in the present study is still relevant.

The analysis of the outcome results at six months did not find statistically significant differences between the three treatments after adjusting by baselines characteristics. Therefore, the superiority of any treatment in clinical terms cannot be established. On the other hand, there were statistically significant differences between the costs for the three interventions.

The combination of outcomes and costs shows that counselling is dominated by the other two options. Therefore, it might be the case that the choice is reduced to that between GET and BUC. GET is more expensive and more effective and, therefore, it is a value judgement as to whether this represents value for money. The CEAC illustrates that the probability that GET is the most cost-effective intervention does not reach a convincing level even when a high level is placed on a clinically significant improvement. If society values such an outcome at £2500 then there is over a 60% likelihood that GET is cost-effective. This uncertainty has been found in previous studies that have analysed the cost-effectiveness of these or alternative interventions [5,6]. Chisholm et al. found that counselling was cheaper than cognitive behavioural therapy (CBT) while in the study by McCrone et al., CBT was also more expensive than the other active therapy (graded exercise). Nevertheless, a clear result as to which intervention was more cost-effective was not reported. These studies were also conducted in primary care with a design very similar to the one presented here although the number of participants in these studies were slightly lower.

A more comprehensive analysis of outcomes trial data [8], including the analysis of effectiveness data at 12 months, found that there were no significant differences in change scores between the three groups at the 6- or 12-month assessments. This analysis also found that, compared to BUC, those treated with graded exercise or counselling therapies were more satisfied at 1 year. Patients with fatigue have reported low levels of satisfaction with diagnosis and management in primary care. Satisfaction is an important and remunerated outcome in the National Health Service, but it is not clear how this can be assessed against increased cost.

### Limitations

First, data collected under experimental conditions may be different from data collected under routine conditions. However, we believe that such bias is minimal in the present study given that patients were drawn from the same practices and the same instruments were used for assessment. Second, reliance on patient self-report might result in some inaccuracies in service use measures. While the schedule used is well developed and has been used in numerous other studies, it may still be the case that for some patients recall was difficult and this would have led to inaccuracies. A number of studies have though suggested that patient recall of service use can be acceptable [23-25]. Another study noted a difference between self-report and administrative records, but pointed out that it was unclear which was more accurate [26]. Third, a unit change on the fatigue scale is difficult to interpret. This is addressed to some extent by using a four-point change to represent clinical significance. However, this is in itself somewhat arbitrary and still presents a challenge for interpretation by those not closely involved in the care of this patient group. Indeed, the self-reported nature of the Chalder Fatigue Scale could prove problematic as it requires respondents to compare themselves with how they were previously (termed as usual) and they may be unsure as to this. The duration of the follow-up period also imposes limitations to the significance of the results of the study. Availability of economic data for the follow-up of a year would have increased the validity and relevance of the results. Our analysis would also benefit from a wider perspective that includes informal care and productivity costs. It was not feasible to use quality-adjusted life years (QALYs) as an outcome measure, as planned in the trial protocol, and this is also a limitation. This was caused by unexpected unavailability of EQ-5D questionnaire data for the six months follow-up. Finally, there were incomplete data for 27 % of the individuals who were randomised. It was decided not to conduct imputations given that there were limited data to do this.

## Conclusion

No clear economic advantage of any of the therapies for the management of patients with chronic fatigue was found in our study. Nevertheless, it does indicate that COUN is *not* cost-effective. With regards to GET, this intervention appears slightly more effective than BUC but this improvement comes at a higher cost. The analyses suggest that the value of a clinically significant improvement needs to be relatively high for GET to be declared the most cost-effective intervention.

## Competing interests

The authors declare that they have no competing interests.

## Authors’ contributions

RSF did the data analysis, participated in the interpretation of the data and prepared the manuscript. PM contributed to the research proposal, reviewed the analysis, participated in the interpretation of the data and participated in the preparation of the manuscript. MH, MK and AND participated in the research proposal and the conduct of the study, and reviewed the manuscript. LR originated the idea for this study, developed the research proposal with MH and MK, led on the conduct of the study, participated in the interpretation of the data and participated in the preparation of the manuscript. All authors have read and approved the final manuscript.

## Pre-publication history

The pre-publication history for this paper can be accessed here:

http://www.biomedcentral.com/1472-6963/12/264/prepub
